# Scaffold Proteins Regulating Extracellular Regulated Kinase Function in Cardiac Hypertrophy and Disease

**DOI:** 10.3389/fphar.2016.00037

**Published:** 2016-02-29

**Authors:** Yan Liang, Farah Sheikh

**Affiliations:** Department of Medicine, University of California-San Diego, La JollaCA, USA

**Keywords:** MAPK and ERK signaling, cardiac myocyte, scaffolding proteins, mouse models, cardiac hypertrophy

## Abstract

The mitogen activated protein kinase (MAPK)-extracellular regulated kinase 1/2 (ERK1/2) pathway is a central downstream signaling pathway that is activated in cardiac muscle cells during mechanical and agonist-mediated hypertrophy. Studies in genetic mouse models deficient in ERK-associated MAPK components pathway have further reinforced a direct role for this pathway in stress-induced cardiac hypertrophy and disease. However, more recent studies have highlighted that these signaling pathways may exert their regulatory functions in a more compartmentalized manner in cardiac muscle. Emerging data has uncovered specific MAPK scaffolding proteins that tether MAPK/ERK signaling specifically at the sarcomere and plasma membrane in cardiac muscle and show that deficiencies in these scaffolding proteins alter ERK activity and phosphorylation, which are then critical in altering the cardiac myocyte response to stress-induced hypertrophy and disease progression. In this review, we provide insights on ERK-associated scaffolding proteins regulating cardiac myofilament function and their impact on cardiac hypertrophy and disease.

## Scaffold Proteins That Regulate Erk-Associated Mitogen Activated Protein Kinase Function: Growing Importance In Cardiac Hypertrophy

The mitogen activated protein kinase (MAPK)/extracellular regulated kinase (ERK) signaling pathway is one of the first blueprints describing how extracellular cues/factors (growth factors and hormones) generate intracellular signals and converge on the nucleus to drive gene expression and a wide range of cellular functions (Pearson et al., 2001). How this single pathway regulates diverse functions and achieves specificity in function in different cell types has been a longstanding question in the field. Studies have suggested that scaffolding proteins are emerging as playing an essential role in regulating the MAPK signaling response (Brown and Sacks, 2009). MAPK scaffold proteins are dynamic entities that (i) directly interact with multiple components of the MAPK signaling complex, (ii) consolidate or sequester protein interactions to physically insulate the MAPK pathway to specific cellular locations as well as (iii) regulate signal strength and stimulus-specific responses to efficiently transmit MAPK signals in a spatiotemporal manner and narrow its actions (Brown and Sacks, 2009). Notable MAPK scaffolds that regulate ERK signaling include, kinase suppressor of Ras (KSR) (Kornfeld et al., 1995; Sundaram and Han, 1995; Therrien et al., 1995), MAPK organizer 1, MORG1 (Vomastek et al., 2004), MEK partner 1, MP1 (Schaeffer et al., 1998), paxillin (Ishibe et al., 2003), β-arrestin (Raabo et al., 1992), and MEKK1 (Karandikar et al., 2000). KSR is one of the best-characterized mammalian MAPK scaffold proteins (Kornfeld et al., 1995). KSR is critical in recruiting the Raf/MEK/ERK complex to the plasma membrane as well as regulating MAPK signaling strength and duration at this location (Brown and Sacks, 2009). Its importance was most notable in *Caenorhabditis elegans* development as well as immune and cancer cells since *in vivo* models deficient in KSR-1 exhibited deficiencies in ERK activation in a stimulus-specific manner, resulting in resistance to inflammatory diseases and Ras-mediated cancers (Nguyen et al., 2002; Lozano et al., 2003; Fusello et al., 2006).

**FIGURE 1:**
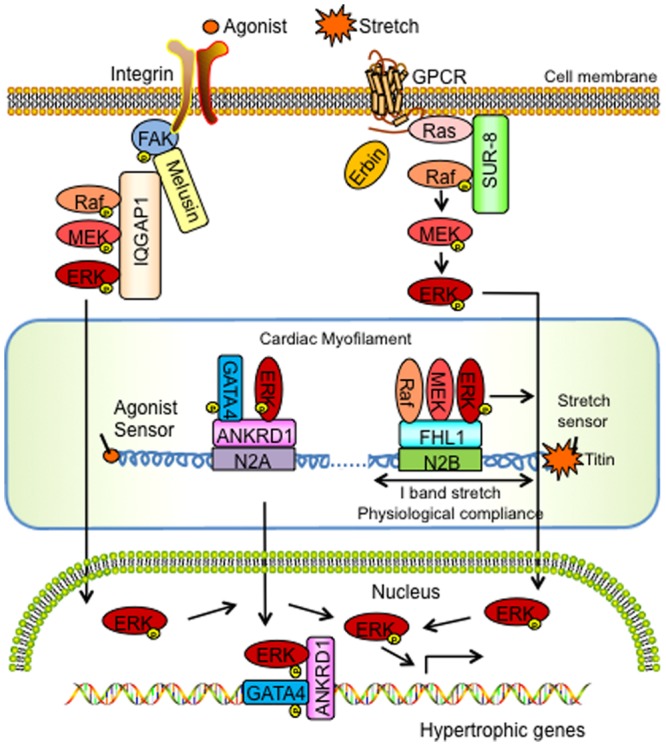
**Schemata of the signaling actions of MAPK scaffolding proteins in cardiac hypertrophy and disease.** ANKRD1, Ankyrin Repeat Domain 1; ERK, extracellular signal-regulated kinase; FAK, focal adhesion kinase; FHL1, Four and a half LIM domain protein-1; GATA4, GATA binding protein-4; GPCR, G Protein Coupled Receptor; IQGAP1, IQ motif-containing guanine-5′-triphosphate hydrolase activating protein; MEK, mitogen-activated protein kinase kinase; SUR-8, Suppressor of Ras-8.

Recently, MAPK scaffold proteins have been identified in the heart and shown to play a pivotal role in pathophysiological cardiac hypertrophy (**Figure 1**). Cardiac hypertrophy is the main way in which cardiomyocytes initially adapt to increased mechanical workload and neurohormonal stimuli to increase cardiac output and compensate for adverse effects on cardiac pump function (Bernardo et al., 2010). Physiological cardiac hypertrophy (i.e., the ‘athlete’s heart’) is characterized by normal cardiac organization and normal or enhanced cardiac function. However, pathological cardiac hypertrophy is a key risk factor for heart failure and is associated with increased interstitial fibrosis, apoptosis, and cardiac dysfunction (McMullen and Jennings, 2007). More recently studies have linked Ras-Raf1-MEK1/2-ERK1/2 signaling pathway in pathological cardiac hypertrophy (Lorenz et al., 2009b). Both constitutive activation of Ras (H-Ras-V12) and overexpression of MEK1 in the mouse heart displayed characteristic features of exaggerated cardiac hypertrophy (Bueno et al., 2000; Zheng et al., 2004). Inhibition of the ERK pathway via dominant negative Raf or cardiac specific deletion of c-raf-1 attenuated pathological cardiac hypertrophy (Yamaguchi et al., 2004; Goldsmith and Dhanasekaran, 2007). The recent identification of a new autophosphorylation site of ERK2 at Thr188 (Thr208 in ERK1) demonstrated a specific role for ERK signaling in the induction of pathological cardiac hypertrophy in response to various hypertrophic stimuli (Lorenz et al., 2009a). Interference with ERK^Thr188^ phosphorylation attenuated ERK-mediated pathological hypertrophy, but not physiological cardiac hypertrophy (Ruppert et al., 2013). More recent efforts have been devoted to understanding how MAPK scaffold proteins regulate ERK signaling strength and duration as well as their role in cardiac muscle in the context of hypertrophy. This review will highlight the subcellular functions and actions of ERK-associated MAPK scaffolds in cardiac muscle and pathological cardiac hypertrophy, with a particular focus on evidence gained from genetic mouse models (**Table 1**).

**Table 1:** Genetic mouse models targeting MAPK scaffold proteins and ERK-mediated signaling in cardiac muscle and their role in cardiac hypertrophy.

MAPK Scaffold protein	Localization	Interactions with MAPK components	Genetic Mouse Model	Cardiac phenotype	Reference
FHL-1	Sarcomere	Scaffolds Raf-1/MEK2/ERK2 at titin N2B	*Fhl1^-/-^*	Blunted and beneficial functional response to pressure overload-induced hypertrophy in adult heart	Sheikh et al., 2008
ANKRD1	Sarcomere	Scaffolds ERK/GATA4 complex at titin N2A	*Ankrd1^-/-^*	Blunted hypertrophic response to chronic phenylephrine infusion in adult heart	Zhong et al., 2015
IQGAP1	Membrane	Scaffolds cRaf-MEK1/2-ERK1/2 via melusin and FAK	*IQGAP1^-/-^*	Impaired cardiac hypertrophic response and increased apoptosis upon prolonged pressure overload in adult heart	Sbroggio et al., 2011b
SUR-8	Membrane	Scaffolds Ras and Raf	*Sur-8^-/-^* and *Sur-8^Δ/flox^; Tie2-Cre*	Early embryonic lethality Late embryonic lethality and cardiac failure	Yi et al., 2010
		Erbin disrupts the Sur-8-Ras-Raf complex	*Erbin^-/-^*	Exaggerated decompensated cardiac hypertrophy and heart failure in response to pressure overload in adult heart	Rachmin et al., 2014

## Myofilament-Associated Scaffolding Proteins that Regulate ERK Function in Cardiac Hypertrophy and Disease

### Four and a Half LIM Domain Protein-1 (FHL1)

Studies from our laboratory have reported FHL1 as a novel ERK associated MAPK scaffold protein that mediates MAPK/ERK signaling responses at the sarcomere during stress-induced cardiac hypertrophy (Sheikh et al., 2008; Raskin et al., 2012). FHL1 is first detected at embryonic day (E) 8.5 in the mouse heart during early development (Chu et al., 2000). Although FHL1 is ubiquitously expressed, it is found to be highly abundant in mammalian striated muscle (Chu et al., 2000). Various studies have shown that FHL1 interacts with multiple binding partners, including structural proteins, signal transducers, transcription regulators, receptors, and channels, to regulate different biological processes (Shathasivam et al., 2010). However, most notably in the heart, FHL1 becomes highly upregulated during hypertrophic conditions in mice and humans (Hwang et al., 1997, 2000; Chu et al., 2000; Lim et al., 2001). Studies in FHL1 knockout (*Fhl1^-/-^*) mice demonstrated that FHL1 is dispensable for survival and basal heart function (Sheikh et al., 2008). However, under conditions of pressure overload, adult *Fhl1^-/-^* hearts displayed a blunted response to cardiac hypertrophy (Sheikh et al., 2008). Most notably, *Fhl1^-/-^* mice subjected to transverse aortic constriction (TAC) maintained cardiac function comparable to sham-operated control mice, and unlike control mice, which exhibited pathological cardiac hypertrophy and dysfunction (Sheikh et al., 2008), highlighting that loss of FHL1 is essential for preservation of cardiac function in pressure overload.

*In vivo* studies performed in transgenic mice overexpressing a constitutively active form of Gαq selectively in the heart, highlighted that FHL1 intersected with Gαq signaling pathways (Sheikh et al., 2008). The Gαq signaling pathway has also been shown to be critical for pressure-overload induced cardiac hypertrophy (D’Angelo et al., 1997; Wettschureck et al., 2001). Interestingly, deletion of *Fhl1* was shown to be sufficient to prevent the cardiomyopathy in Gαq overexpressing transgenic mice (Sheikh et al., 2008). Yeast two-hybrid and co-immunoprecipitation based assays further identified that FHL1 could directly interact with MAPK components, Raf1, MEK2, and ERK2 *in vitro* and in *in vivo* (Sheikh et al., 2008). Immunofluorescence microscopy studies further showed that the FHL1/Raf-1/ MEK2/ERK2 complex all localized to the sarcomeric I band, and thus highlighted a MAPK scaffolding function for FHL1 at the titin N2B region of the sarcomere (Sheikh et al., 2008).

In terms of downstream effects on MAPK/ERK signaling, FHL1 overexpression was sufficient to increase ERK1/2 phosphorylation in cardiomyocytes *in vitro* (Sheikh et al., 2008). *In vivo* studies performed in FHL1 deficient settings such as TAC subjected *Fhl1^-/-^* adult hearts as well as double *Fhl1^-/-^*/Gαq constitutively overexpressing adult hearts demonstrated a blunted response to ERK1/2 phosphorylation, suggesting that FHL1 can positively regulate Raf-1/MEK/ERK-mediated signaling in a Gαq stimulus-specific manner (Sheikh et al., 2008). Convergence of these pathways to titin N2B function was shown in isolated adult *Fhl1^-/-^* cardiac muscles subjected to a maximum extension of 15–20% stretch (Sheikh et al., 2008; Raskin et al., 2012). Stretched *Fhl1^-/-^* cardiac muscles displayed a loss of upregulation of Elk-1 mRNA (transcriptional target of ERK1/2 signaling) and diastolic stress when compared with controls, further demonstrating an impact of loss of FHL1 and MAPK-ERK signaling on cardiac muscle compliance (Sheikh et al., 2008; Raskin et al., 2012).

Phosphorylation of titin N2B has been reported to be pivotal in mediating muscle compliance in cardiomyocytes (Fukuda et al., 2005). Interestingly, titin N2B isoform expression was increased in adult *Fhl1^-/-^* cardiac muscles and these muscles were also found to be insensitive to titin N2B phosphorylation-mediated diastolic reduction, as they intrinsically exhibited functions associated with maximal titin N2B phosphorylation (Raskin et al., 2012). *In vitro* studies demonstrated that ERK2 can directly phosphorylate titin N2B (Ser-3873, Ser-3915, Ser-3965) and that FHL1 functioned to interfere with ERK2- mediated titin N2B phosphorylation (Raskin et al., 2012). Thus, in FHL1 deficient settings titin N2B phosphorylation was thought to be hyper or maximally phosphorylated correlating with increased compliance. These studies lead to a model whereby FHL1 acts to scaffold MAPK components at titin N2B in order to regulate cardiac muscle compliance (via titin N2B phosphorylation) and hypertrophic signaling under conditions of stretch/hypertrophic stress/Gq (Raskin et al., 2012). Interestingly in FHL1 deficient settings, a unique role for the MAPK component, ERK2, was identified as it was no longer tethered to titin N2B but instead “open” to directly phosphorylate titin N2B to increase compliance of cardiac muscle as opposed to being sequestered to participate in hypertrophic signaling (Raskin et al., 2012). These results suggest that FHL1 functions as a MAPK scaffold by directly interacting with Raf-1/MEK2/ ERK2 to physically insulate the MAPK pathway to the titin N2B region of the sarcomere in a Gq stimulus-specific manner and efficiently transmit MAPK signals to regulate cardiac hypertrophy (**Figure 1**).

### Ankyrin Repeat Domain 1 (ANKRD1)

Recent studies also suggested that ANKRD1 [also known as cardiac adriamycin responsive protein (CARP)] is an ERK associated protein that mediates ERK signaling responses at the sarcomere (titin N2A) during phenylephrine-induced hypertrophy (Zhong et al., 2015). ANKRD1 is a transcriptional cofactor and highly expressed in embryonic cardiomyocytes (Mikhailov and Torrado, 2008). ANKRD1 was shown to negatively regulate gene expression of cardiac-specific genes in fetal cardiomyocytes *in vitro* (Jeyaseelan et al., 1997; Zou et al., 1997; Kuo et al., 1999). In adult cardiac muscle ANKRD1 expression is developmentally downregulated but was reported to increase in animal models of cardiac hypertrophy and human patients with various forms of cardiomyopathy leading to heart failure (Arber et al., 1997; Kuo et al., 1999; Aihara et al., 2000; Maeda et al., 2002; Zolk et al., 2003). Genetic mutations in ANKRD1 were also found to be causative for hypertrophic and dilated cardiomyopathy (Arimura et al., 2009; Duboscq-Bidot et al., 2009; Moulik et al., 2009). Based on the direct interaction between ANKRD1 and titin N2A region, it was thought that ANKRD1 may play a critical role in linking stretch-induced signaling pathways and muscle gene expression (Miller et al., 2003). However, *in vivo* studies performed in *Ankrd1* knockout mice (*Ankrd1^-/-^*) highlighted an indispensable role for ANKRD1 in the heart at basal conditions (Bang et al., 2014). In addition, *Ankrd1^-/-^* mice subjected to pressure overload via TAC, were also able to maintain a normal cardiac hypertrophic response, similar to control mice (Bang et al., 2014). These results were supported by triple (*Ankrd1/Ankrd2/Ankrd23*) *MARP* knockout mice, which also mounted a normal cardiac hypertrophic response to TAC similar to controls (Bang et al., 2014).

*In vitro* and *in vivo* studies have suggested that ANKRD1 may be coupled to the α-adrenergic associated hypertrophic pathway (Maeda et al., 2002; Zhong et al., 2015). *Ankrd1^-/-^* mouse hearts and *Ankrd1* knockdown by shRNA was shown to attenuate the hypertrophic response to the α-adrenergic agonist, phenylephrine (PE) (Zhong et al., 2015). Specifically, PE infused *Ankrd1^-/-^* mice displayed reduced heart size similar to vehicle controls (Zhong et al., 2015). Co-immunoprecipitation and immunostaining studies showed that ANKRD1, ERK, and GATA4 formed a complex at the sarcomeric I band and nucleus (Zhong et al., 2015). Cardiomyocytes showed that cardiomyocytes transfected with *Ankrd1* siRNA resulted in diffuse cytoplasmic staining of both ERK1/2 and GATA4, suggesting that ANKRD1 may serve as a scaffolding protein to anchor ERK and GATA4 (Zhong et al., 2015). GATA4, which is a zinc finger transcription factor, plays a pivotal role in cardiac development and cardiac hypertrophy (Oka et al., 2006). Recent studies highlight that ERK phosphorylates GATA4 at S105, leading to enhanced GATA4-DNA binding and activation of ERK1/2-induced cardiac hypertrophy (van Berlo et al., 2011). These findings suggested that ANKRD1 acts as a scaffold to enhance GATA4 phosphorylation via ERK1/2 in the sarcomere and that the ANKRD1-ERK-GATA4 complex is translocated to the nucleus upon PE stimulation to regulate hypertrophic responses (**Figure 1**). *In vitro* findings support this mechanism as cardiomyocytes transfected with *Ankrd1* siRNA were shown to have an attenuated increase in PE-mediated pERK1/2 and pGATA4 as well as signals related to the nuclear ERK1/2-GATA4 complex (Zhong et al., 2015).

## Plasma Membrane-Associated Scaffolding Proteins that Regulate ERK Function in Cardiac Hypertrophy and Disease

### IQ Motif-Containing GTPase Activating Protein (IQGAP1)

IQGAP1 also acts as a membrane-bound MAPK scaffold to regulate responses associated with cardiac hypertrophy. IQGAP1 is a highly conserved cytoplasmic scaffold protein that is ubiquitously expressed (Weissbach et al., 1994). It interacts with many binding partners and is involved in cytoskeleton, cell-cell adhesion, protein trafficking, and cell signaling (Smith et al., 2015). *In vivo* studies performed in *IQGAP1* null mice, demonstrated a dispensable role for IQGAP1 in heart function at basal conditions (Sbroggio et al., 2011b). Also, during chronic pressure overload induced by TAC, *IQGAP1* null mice initially displayed a compensatory hypertrophic response identical to control mice. However, upon prolonged pressure overload, *IQGAP1* null mice displayed unfavorable cardiac remodeling, contractile dysfunction, impaired cardiac hypertrophy, and increased apoptosis when compared with control mice (Sbroggio et al., 2011b). The deletion of *IQGAP1* did not affect global MEK-ERK1/2 signaling in the heart, but selectively impaired the second wave of ERK1/2 activation which peaked at 4 days in response to pressure overload (Sbroggio et al., 2011b).

Mechanisms underlying the effects of IQGAP1 in cardiac hypertrophy may be due to a direct interaction with melusin. Melusin is a muscle specific protein that binds to the cytoplasmic domain of β1 integrin, thought to act as a mechanical stretch sensor (Brancaccio et al., 1999). Melusin expression increases with various hypertrophic stimuli (cyclic mechanical stretch, angiotensin-II, endothelin-1, and phenelyphrine) in neonatal ventricular cardiomyocytes *in vitro* (Aro et al., 2013), suggesting a direct role in cardiac hypertrophy. *In vivo* studies performed with *Melusin*-null mice subjected to pressure overload demonstrated reduced left ventricular hypertrophy, accelerated development of dilated cardiomyopathy and contractile dysfunction (Brancaccio et al., 2003). Biphasic effects of melusin were demonstrated via *in vivo* studies involving transgenic mice that selectively overexpressed *melusin* in the heart (De Acetis et al., 2005). Cardiac-specific overexpression of *melusin* induced cardiomyocyte hypertrophy during basal conditions and retained compensatory hypertrophy during acute pressure overload; however, the transition toward heart failure through activation of ERK phosphorylation was prevented in response to prolonged pressure overload (De Acetis et al., 2005). Studies performed using transgenic mice overexpressing melusin also demonstrated cardioprotective effects of melusin during myocardial infarction as well as ERK-dependent cardioprotective effects of melusin during ischemia-reperfusion injury (Penna et al., 2014; Unsold et al., 2014).

Cross-talk between IQGAP1 and melusin was shown in hearts of double *IQGAP1* null/*melusin* overexpressing mice, which demonstrated that the absence of *IQGAP1* abrogated melusin-induced ERK1/2 hyper-phophorylation in both basal conditions and in response to pressure overload (Sbroggio et al., 2011a). Co-immunoprecipitation studies in the heart demonstrated that melusin, IQGAP1 and cRaf-MEK1/2-ERK1/2 formed a supercomplex with focal adhesion kinase (FAK) at the membrane (Sbroggio et al., 2011a).

Focal adhesion kinase is a well-known downstream target of integrin, which is activated by biomechanical stress and agonists as well as mediates ERK1/2 pathway activation (Lal et al., 2007). Studies performed in FAK deficient settings showed that depletion of *FAK* does not affect the phenotype of non-stimulated cardiomyocytes, but blocked the up-regulation of hypertrophic gene markers after pressure overload (Marin et al., 2008). Furthermore, *in vivo* studies showed that depletion of global myocardial FAK by RNA interference or cardiac-specific *FAK* knockout mice prevented left ventricular hypertrophy and the deterioration of cardiac structure after TAC and angiotensin II infusion (DiMichele et al., 2006; Clemente et al., 2007). These studies further suggested an important requirement for FAK in melusin-dependent ERK activation and cardiomyocyte hypertrophy during pressure overload. Cardiomyocytes treated with the FAK inhibitor (PF573228) or knockdown (*FAK* shRNA) demonstrated an abolishment of melusin-dependent hypertrophy and reduced ERK1/2 phosphorylation (Sbroggio et al., 2011a). Thus, FAK, melusin and IQGAP1 were all shown to be required for ERK1/2 activation in response to pressure overload (Sbroggio et al., 2011a). These data altogether suggested a model whereby IQGAP1 scaffolds MAPK components (c-Raf, MEK1/2 and ERK1/2) at the plasma membrane via Melusin and FAK to form a supercomplex to regulate MAPK-ERK signaling and cardiac hypertrophy (**Figure 1**).

### Suppressor of Ras-8 or Shoc2 (SUR-8)

Suppressor of ras-8, a conserved leucine-rich repeat protein, was identified as a positive regulator of Ras-mediated signal transduction in *C. elegans* (Sieburth et al., 1998). Studies show that SUR-8 functions as a MAPK scaffold that forms a ternary complex with Ras and Raf, which then specifically enhances Ras-induced ERK activation by facilitating the interaction between Ras and Raf, without any effect on AKT or JNK activity (Li et al., 2000). *Sur-8* conventional knockout mice were embryonic lethal at early stages (around E8.5) of mouse development (Yi et al., 2010). Endothelial-specific Sur-8 knockout mice displayed embryonic lethality at later stages of mouse development as well as multiple cardiac defects (Yi et al., 2010).

Although the mechanisms underlying how SUR-8 regulates MAPK signaling in the heart are not well understood, recent data suggests an intersection with Erbin and cardiac hypertrophy. Erbin is a LAP [leucine-rich repeat (LRR) and PDZ domain] family protein (Dillon et al., 2002). Recent studies demonstrated that LRR- and PDZ-domain-containing proteins play a key role in assembling protein complexes for signal transduction, suggesting a signaling role for Erbin (Birrane et al., 2003). Erbin has been reported to inhibit Ras-mediated activation of the MAPK pathway via an effect on SUR-8. Specifically, the LRR domain of Erbin was shown to bind to SUR-8 and inhibit the interaction of SUR-8 with Ras and Raf (Dai et al., 2006). Interestingly, Erbin inhibited Raf activation, but had no effect on Ras activation (Dai et al., 2006). Furthermore, *Erbin* overexpression was sufficient to inhibit the interaction between SUR-8 and Ras and Raf (Dai et al., 2006). Suppression of *Erbin* by shRNA increased the interaction of SUR-8 with Ras and Raf, resulting in enhanced Raf and ERK activation (Dai et al., 2006). Co-immunoprecipitation studies showed that Erbin can form an endogenous complex with SUR-8 in adult mouse hearts and cardiomyocytes and that this interaction was lost in Erbin deficient (*Erbin^-/-^*) hearts, which now resulted in p-Raf binding to SUR-8 and thus, ERK1/2 phosphorylation (Rachmin et al., 2014). Interestingly, Erbin expression is downregulated in adult mouse hearts subjected to hypertrophic agonists or TAC (Rachmin et al., 2014). Studies performed in *Erbin^-/-^* mice demonstrated that Erbin is dispensable for the heart during basal conditions; however, when these mice were subjected to pressure overload they displayed an exaggerated decompensated cardiac hypertrophy and failure when compared with controls (Rachmin et al., 2014). In terms of MAPK-ERK signaling, *Erbin^-/-^* mouse hearts exhibited a twofold increase in p-ERK when compared to controls at baseline and this effect was even more pronounced (threefold) under hypertrophic conditions (Rachmin et al., 2014). These data altogether suggested that Erbin is a negative regulator of the MAPK scaffold, SUR-8, and can disrupt the Sur-8-Raf-Ras complex to regulate cardiac hypertrophic signaling (Rachmin et al., 2014) (**Figure 1**).

## Conclusion and Future Directions

Mitogen activated protein kinase scaffold proteins are emerging as important players in shaping and localizing the MAPK-ERK signaling response in cardiac muscle in order to simplify the response to distinct hypertrophic stimuli (e. g., pressure overload, Gαq, phenylephrine, etc.). Interestingly, loss of function studies in mouse models deficient in MAPK scaffold proteins bound to the sarcomere, such as FHL1 and ANKRD1, suggest a detrimental role in Gαq and phenylephrine-induced cardiac hypertrophy, respectively, while studies on MAPK scaffold proteins bound to the plasma membrane, such as IQGAP1 and Sur-8, suggest a protective role in mechanical induced-cardiac hypertrophy. These studies highlight that targeting distinct scaffolds may allow for more specific MAPK signaling modulation in pathological states associated with cardiac hypertrophy. Given the complexity and pleiotropic actions of the MAPK pathway in cardiac muscle, future studies should also be directed at determining (i) whether other hypertrophic associated MAPK scaffolds existed in cardiac muscle, (ii) how MAPK scaffolds insulated themselves from one another so that cardiac hypertrophic signaling is stringently regulated and (iii) how MAPK scaffolds regulated their hypertrophic function in cardiac muscle in a spatiotemporal manner. Studies focused on developing selective compounds that target these scaffolds may also provide a means to harness MAPK signaling to clinically intervene with the deleterious effects associated with cardiac hypertrophy and failure.

## Author Contributions

YL contributed to drafting the content, figure, and table in the review article. FS contributed to the design as well as finalizing the content, figure, and table in the review article.

## Conflict of Interest Statement

The authors declare that the research was conducted in the absence of any commercial or financial relationships that could be construed as a potential conflict of interest.
